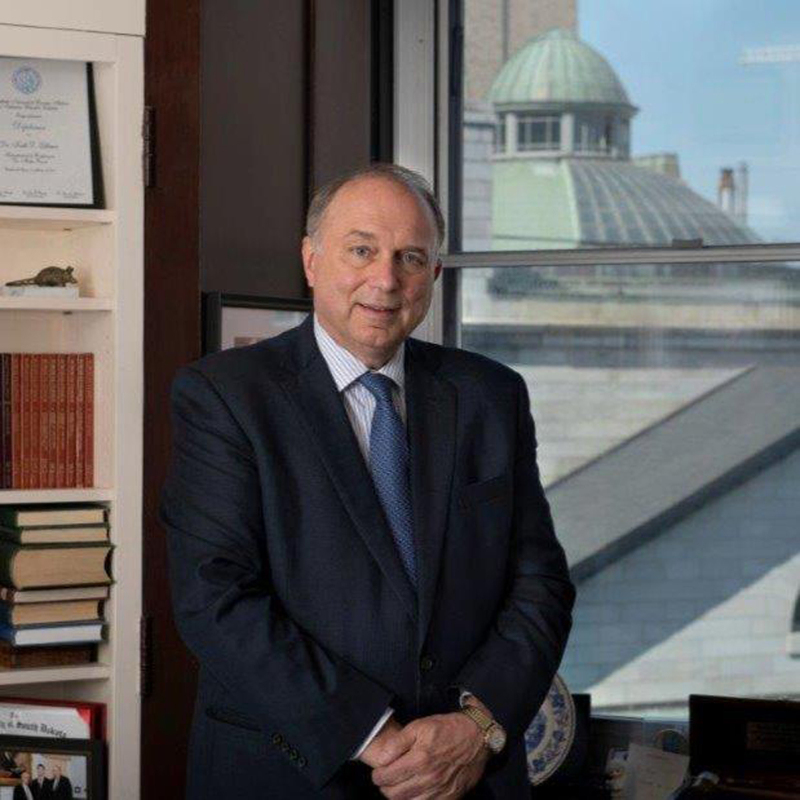# Thank You, Dr. Keith Lillemoe, for Your Service to *Annals of Surgery* and *Annals of Surgery Open*

**DOI:** 10.1097/AS9.0000000000000574

**Published:** 2025-05-06

**Authors:** Justin B. Dimick, Luke M. Funk

**Affiliations:** From the * Department of Surgery, University of Michigan, Ann Arbor, MI; † Department of Surgery, University of Wisconsin-Madison, Madison, WI; ‡ Department of Surgery, William S. Middleton VA, Madison, WI.

Larry Bird, the legendary Boston Celtics forward, once said, “*I’ve got a theory that if you give 100% all of the time, somehow things will work out in the end*.” Few embody that level of commitment, consistency, and excellence more than Dr. Keith D. Lillemoe, who served as Editor-in-Chief of *Annals of Surgery* from 2011 to 2025, and as the founding Editor-in-Chief of *Annals of Surgery Open* from 2020 to 2025.

Those of us who have worked closely with Dr. Lillemoe as part of both journals wish to express our deepest gratitude and admiration for all that he has done during his remarkable tenure. Over the past 14 years, *Annals of Surgery* has grown and evolved in extraordinary ways. Under Dr. Lillemoe’s leadership at *Annals*, the number of submissions has grown from 1600 per year to nearly 2800 per year; citations have surged during Dr. Lillemoe’s 14-year tenure, with *Annals* consistently ranking as the most highly cited surgical journal each year; and the journal’s impact factor has increased substantially, peaking at 12.97 in 2021—solidifying its place as the leading surgical journal in the world.

Even more impressive than these metrics, however, is Dr. Lillemoe’s commitment to supporting surgeon authors and using *Annals* as a platform to foster clinical excellence in surgery. He has upheld the rigorous standards and scientific integrity that define *Annals*, while also fostering an environment that is fair, thoughtful, and supportive to authors, reviewers, and editors alike.

In 2020, Dr. Lillemoe launched *Annals of Surgery Open* to meet the growing demand for high-quality, accessible surgical scholarship. His vision and guidance led to its successful indexing in PubMed Central and a rapidly growing readership and author base. This new journal reflects Dr. Lillemoe’s forward-thinking approach and his unwavering commitment to advancing the field of surgery through the dissemination of knowledge.

Dr. Lillemoe’s editorial legacy is one of scholarly excellence, inclusive leadership, and a deep belief in the transformative power of surgical science. He has raised the bar for what it means to be an Editor-in-Chief, and we are honored to build on the strong foundation he has created.

We wish Dr. Lillemoe all the best in this next chapter and are grateful that he will continue to serve the journals in an advisory capacity.

Thank you, Keith, for 14 exceptional years at *Annals of Surgery* and for launching and leading *Annals of Surgery Open* with vision and dedication.

**Figure:**